# A Case of Outpatient Hysterectomy in the Setting of Gitelman Syndrome

**DOI:** 10.7759/cureus.12129

**Published:** 2020-12-17

**Authors:** Duminda S Siripala, Leah Bagiardi, Emad Mikhail

**Affiliations:** 1 Nephrology, University of Pittsburgh Medical Center Altoona, Altoona, USA; 2 Obstetrics and Gynecology, University of South Florida Morsani College of Medicine, Tampa, USA

**Keywords:** gitelman syndrome, perioperative, hysterectomy, electrolyte, anaesthetic

## Abstract

Gitelman syndrome is a rare autosomal recessive disorder involving a defect in the sodium-chloride cotransporter, which is expressed in the apical membrane of the distal convoluted tubule. Electrolyte abnormalities commonly occur in patients with Gitelman syndrome as a result, including hypokalaemia, hypomagnesemia, and metabolic alkalosis. As a result, the disorder may present with various clinical manifestations, including fatigue, weakness, muscle tetany, facial paresthesias, and a predisposition to the development of various ventricular arrhythmias. As a result, the perioperative management of patients with this disorder presents unique challenges with regard to fluid and electrolyte management and the prevention and management of potential arrhythmias. In addition, the pharmacology of various anesthetics may present additional complexity with regard to perioperative management in this particular patient population. The following case presentation of a 42-year-old female with Gitelman syndrome undergoing elective outpatient hysterectomy for suspected endometriosis serves to illustrate the challenges that arise with regard to perioperative management in this particular patient population and demonstrates how they may be addressed.

## Introduction

Gitelman syndrome is an autosomal recessive disorder that causes a defect in the sodium chloride cotransporter (NCCT), which is expressed in the apical membrane of the distal convoluted tubule of the kidney [1-3]. It is believed that loss of function mutations in the SLC12A3 gene, which encodes the NCCT transporter, are responsible for the majority of cases for the development of Gitelman syndrome. More than 140 loss of function mutations have been identified in the SLC12A3 gene in patients with Gitelman syndrome, although a minority of patients with the disease has mutations in the CLCNKB gene, which encodes the renal chloride channel CIC-Kb, found in the basolateral membrane of cells of the thick ascending limb of the loop of Henle [4]. As a result of the impaired expression of NCCT, sodium reabsorption is impaired, leading to hypokalemia, hypomagnesemia, hypocalciuria, and metabolic alkalosis [2-3].

Gitelman syndrome is distinguished from Bartter’s syndrome, another salt-wasting tubulopathy that involves the thick ascending limb of the loop of Henle, by the fact that Gitelman syndrome typically presents with hypomagnesemia, whereas hypomagnesemia is less common in patients with Bartter’s syndrome, and while hypocalciuria generally occurs with Gitelman syndrome, calcium wasting is more likely to occur with Bartter’s syndrome. It is often said that Bartter’s syndrome mimics the mechanism of action of a loop diuretic, such as lasix or Bumex, whereas Gitelman syndrome mimics the mechanism of action of a thiazide diuretic [2-3]. The mechanism by which this occurs involves the wasting of sodium and chloride due to the fact that reabsorption cannot occur at the distal convoluted tubule. As a result, the activation of the renin-angiotensin-aldosterone (RAA) axis occurs. While activation of the RAA axis allows more distal sodium reabsorption at the collecting duct, potassium and H+ excretion occur in exchange for sodium reabsorption, thus leading to hypokalaemic metabolic alkalosis. Hypomagnesemia occurs in Gitelman syndrome as well, for several possible reasons. It is suspected that the downregulation of the epithelial magnesium channel, TRPMC, is responsible for magnesium wasting. Also, apoptotic and/or histologic changes that occur in the distal convoluted tubule with Gitelman syndrome may impair magnesium reabsorption [1-3].

Common clinical manifestations of Gitelman syndrome include muscle tetany and facial paraesthesias as a result of hypomagnesemia, particularly in cases where additional hypomagnesemia occurs in cases of fever, vomiting, and diarrhea. Some patients also experience severe fatigue, which may impair daily activity. Some patients with Gitelman syndrome may experience growth delay if they have severe hypokalemia and hypomagnesemia. In addition, patients with Gitelman syndrome may develop chondrocalcinosis as a result of chronic hypomagnesemia, leading to swelling, heat, and tenderness of the affected joints. Patients with Gitelman syndrome also experience more salt cravings, generalized weakness and dizziness, nocturia, hypotension, and polydipsia in comparison to individuals without the disorder. Most likely, this is due to increased salt and water losses. Many patients with Gitelman syndrome also develop muscle weakness and cramps, probably as a result of hypokalemia. In addition, patients with Gitelman syndrome may be at a higher risk for the development of ventricular arrhythmias as a result of hypokalemia and hypomagnesemia. Patients with Gitelman syndrome will, in 50% of cases, present with mild to moderate QT prolongation as an EKG finding [4].

Given the potential sequelae that may occur with Gitelman syndrome, patients with this disease present unique challenges with regard to the management in the perioperative setting. The following case presentation of a patient with Gitelman syndrome undergoing outpatient hysterectomy for the treatment of endometriosis serves to illustrate this point.

## Case presentation

The patient is a 42-year-old gravida 0 with a known history of Gitelman Syndrome. She was being managed by nephrology and had a mediport in place for home intravenous (IV) electrolyte replacement. She was receiving IV magnesium sulfate 6 g and potassium chloride 80 mEq nightly infusion over 9.5 hours in 500 mL normal saline. She was also taking potassium chloride 20 mEq PO four times daily with an additional 10 mEq as needed for symptomatic hypokalemia.

She initially presented for chronic pelvic pain, menorrhagia, and dysmenorrhea. She had previously attempted medical management with continuous oral contraceptive pills (OCPs) without improvement in her symptoms. Her history, physical examination, and workup were indicative of endometriosis and, considering she had previously failed medical management, a decision was made to proceed with surgical management. The patient elected to proceed with a total laparoscopic hysterectomy and bilateral salpingectomy.

Considering her history of Gitelman syndrome, the patient was seen by nephrology and cardiology for clearance prior to surgical intervention. She had an echocardiogram, which was unremarkable. She had also reported a history of postoperative hallucinations, cognitive dysfunction, and hypokalemia. She was evaluated by anesthesiology prior to her procedure, with plans for close electrolyte monitoring before, during, and after the case. On the day of the procedure, she had a starting potassium of 5.1 and magnesium of 2.9. However, the preoperative potassium was suspected to be falsely elevated, as two tourniquets were used in order to obtain the blood sample.

The patient underwent a total laparoscopic hysterectomy, laparoscopic bilateral salpingectomy, and cystoscopy. Throughout the case, she had arterial blood gas and electrolyte monitoring approximately every 20-40 minutes with repletion as needed. Intraoperatively, she received a total of 15 mEq potassium and 1 g of magnesium for hypokalemia and hypomagnesemia, respectively. Immediate postoperative labs showed a potassium of 4.0 and magnesium of 2.0. Postoperatively, the patient experienced mild right eye heaviness and nausea, which she reported can be indicative of hypomagnesemia and hypocalcemia. Labs were drawn at that time, showing potassium, magnesium, and calcium levels all within the normal range. At that time, the patient requested to take her home PO potassium. After taking this medication, she was feeling well and meeting all postoperative discharge milestones. She was discharged home four hours postoperatively.

## Discussion

Special considerations must be followed with regard to the perioperative management of patients with Gitelman syndrome. Regarding perioperative management, there are guidelines for electrolyte concentrations that have been determined safe in patients undergoing surgical procedures. It is recommended, for instance, in Gitelman syndrome, that for patients in the perioperative setting, if the serum magnesium concentration is greater than or equal to 0.5 mmol/liter, the serum potassium level should be at a minimum of 3 mmol/liter. If the serum magnesium level, however, is less than 0.5 mmol/liter, the threshold for the serum potassium level is higher, at 3.3 mmol/liter [1]. Although there is limited evidence regarding the potential for hypomagnesemia to cause arrhythmia alone, hypomagnesemia can increase the risk of arrhythmia in the setting of hypokalemia. Therefore, the potassium and magnesium levels are both considered when determining the acceptable lower limit that is safe to proceed with surgery. Also, carefully monitored replacement of potassium and/or magnesium, including during surgery, is recommended and continuous ECG monitoring should be available, including during recovery [1]. It appears that guidelines with regard to appropriate electrolyte levels were followed when electing to proceed with surgery in the case discussed above. Although, as there is a possibility that pseudohyperkalaemia may have occurred as a result of using two tourniquets to draw the preoperative serum potassium level, repeating the preoperative serum potassium level would have been well-advised.

The guidelines for perioperative management also recommend electrolyte monitoring immediately postoperatively, in particular for major surgeries such as abdominal hysterectomy, lumbar discectomy, or thyroidectomy [1]. This is most likely because electrolyte shifts are more likely to occur during major procedures. Most likely, this is due to catecholamine-induced stress responses, which have been found to cause potassium shifts. Frequently, hypokalaemia will occur during procedures such as direct laryngoscopy and endotracheal intubation as a result of catecholamine-induced stress responses [5]. In addition, procedures such as laryngoscopy, endotracheal intubation, and painful stimuli during surgery have been found to exacerbate QT prolongation [6]. As has been noted previously, patients with Gitelman syndrome tend to be more predisposed to the development of QT prolongation, which is most likely due to underlying hypokalemia. It is not unreasonable to presume that the potassium shifts caused by noxious stimuli during surgery are a causative factor in the development of QT prolongation. Therefore, in patients with Gitelman syndrome, it is important to ensure that adequate depth of anesthesia is maintained in order to prevent the development of catecholamine-induced stress responses, potentially leading to hypokalemia and QT prolongation. Close monitoring of electrolytes and cardiac rhythms appears to have been performed in the case discussed above, especially as the electrolytes were monitored every 20-40 minutes intraoperatively and were replenished as needed.

Also, caution should be exercised when considering the use of volatile halogenated anesthetics, as these may potentially cause QT prolongation. Alternatives that may be considered include total intravenous anesthesia (TIVA) with propofol infusion [6]. Also, as patients with Gitelman syndrome are frequently hypomagnesemic, they are also more at risk for the development of torsades de pointes. Therefore, caution should be exercised when considering the use of medications such as macrolides, antihistamines, antiarrhythmics, and antipsychotics in the perioperative setting, as torsades de pointes is a potential adverse effect that may occur with those drugs [6]. In addition, respiratory alkalosis may potentially cause hypokalaemia [6]. Therefore, anesthesiologists should closely adjust ventilatory settings and parameters in order to avoid this in patients with Gitelman syndrome, as this patient population is more predisposed to the development of hypokalemia. Other drugs that may be considered in the perioperative setting in order to prevent the development of hypokalemia and hypomagnesemia may include amiloride, which blocks the sodium reabsorption channel (ENac) in the principal cell of the collecting duct, thus preventing the excretion of potassium and H+ in exchange for sodium and potassium-sparing agents such as spironolactone [4]. In patients with Gitelman syndrome, postanesthesia care should occur in a closely monitored setting for 24-48 hours or until the patient is clinically stable with normal potassium and magnesium levels. If cardiac arrhythmias develop in patients with Gitelman syndrome postoperatively, serum potassium, calcium, and magnesium levels should be immediately checked and corrected if necessary [5]. The patient in the case mentioned above did have normal potassium and magnesium levels immediately postoperatively and appeared to be clinically stable. However, repeat serum potassium, calcium, and magnesium levels would have been advisable prior to discharge four hours later.

## Conclusions

In summary, the perioperative management of patients with Gitelman's syndrome is complicated by many factors, including potential risk for the development of arrhythmias, the impact of anesthetics and other perioperative medications upon electrolyte status, risk for the potentiation of arrhythmias, and the effect of various surgical stressors on the development of electrolyte abnormalities and arrhythmias. Successful management of this patient population in the perioperative setting would most likely benefit from a team approach, involving collaboration between anesthesiologists, surgeons, general internal medicine, and nephrology. It is our opinion that nephrology consultation should always be obtained in the event a patient with Gitelman syndrome undergoes surgery. Perioperative management in patients with Gitelman syndrome carries certain special considerations that are unique to this particular population.

## References

[1] Gallagher H, Soar J, Tomson C (2016). New guideline for perioperative management of people with inherited salt-wasting alkaloses. Br J Anaesthesia.

[2] Gross P, Heduschka P (2015). Inherited disorders of sodium and water handling. Comprehensive Clinical Nephrology.

[3] Pham P-ChiT, Pham P-TT (2017). Acid-base/potassium. Nephrology and Hypertension Board Review.

[4] Knoers NV, Lentchenko EM (2008). Gitelman syndrome. Orphanet J Rare Dis.

[5] Bissonette B, Luginbuehl J, Marciniak B, Dalens J (2006). Gitelman syndrome. Syndromes: Rapid Recognition and Perioperative Implications.

[6] Shah RB, Shah VR, Parikh GP, Vora KS (2016). Anaesthesia in a patient with Gitelman syndrome. J Anaesthesiol Clin Pharmacol.

